# Applications of the Kirkpatrick Model in Post-secondary Health Sciences Education: A Scoping Review

**DOI:** 10.34172/ijhpm.8857

**Published:** 2026-04-06

**Authors:** Natasha L. Gallant, Elizabeth Oddone Paolucci, Chelsea L. Russill, Ray Jewett, Chantelle Recsky, Katherine Ford, Dina Idriss-Wheeler, Victrine Tseung, Hina Ansari, Zeenat Ladak, Aida Fernandes, Deborah A. Marshall

**Affiliations:** ^1^Department of Psychology, Faculty of Arts, University of Regina, Regina, SK, Canada.; ^2^Departments of Community Health Sciences and Surgery, Cumming School of Medicine, University of Calgary, Calgary, AB, Canada.; ^3^Department of Geography and Planning, University of Toronto, Toronto, ON, Canada.; ^4^Ontario Health, Toronto, ON, Canada.; ^5^School of Nursing, Faculty of Applied Sciences, University of British Columbia, Vancouver, BC, Canada.; ^6^Department of Kinesiology and Health Sciences, Faculty of Health, University of Waterloo, Waterloo, ON, Canada.; ^7^Interdisciplinary School of Health Sciences, Faculty of Health Sciences, University of Ottawa, Ottawa, ON, Canada.; ^8^School of Rehabilitation Science, Faculty of Health Sciences, McMaster University, Hamilton, ON, Canada; ^9^MAP Centre for Urban Health Solutions, Li Ka Shing Knowledge Institute, St Michael’s Hospital, Toronto, ON, Canada.; ^10^Department of Applied Psychology & Human Development, Ontario Institute for Studies in Education, University of Toronto, Toronto, ON, Canada.; ^11^IMAGINE Network SPOR, McMaster University, Hamilton, ON, Canada.; ^12^Departments of Community Health Sciences and Medicine, Cumming School of Medicine, University of Calgary, Calgary, AB, Canada.

**Keywords:** Education, Evaluation, Experiential Learning, Healthcare Professions, Health Systems, Impact

## Abstract

**Background:**

The Kirkpatrick model is commonly used as a systematic approach to evaluate training programs, although its application to health sciences experiential learning programs is not well-established. To inform the use of the Kirkpatrick model in the evaluation of the Canadian Institutes of Health Research’s (CIHR’s) Health System Impact Fellowship National Cohort Training Program (HSIF NCTP), we examined its application in post-secondary health sciences programs.

**Methods:**

Using the Joanna Briggs Institute’s updated methodology for scoping reviews, we searched CINAHL, EMBASE, ERIC, MEDLINE, PsycINFO, and Web of Science for studies published from 2017 to 2023 that focused on health sciences experiential learning programs held at universities and reported on at least one level of the Kirkpatrick model (ie, reaction, learning, behavior, results). We extracted data on study characteristics and reported outcomes for each of the Kirkpatrick model levels.

**Results:**

After deduplication, we screened 755 titles and abstracts, we reviewed 97 full texts, and we included 34 studies in our scoping review. Many studies reported outcomes at the reaction or learning levels followed by the behaviour and results levels. Across levels, despite identifying several areas of improvement, learners typically reported favourable perceptions, increased confidence and knowledge, improved performance, and organizational improvements.

**Conclusion:**

The Kirkpatrick model is a widely used and highly adaptable evaluation model that has been successfully used to evaluate a range of post-secondary health sciences programs. Despite its wide use, evaluators using the Kirkpatrick model should use more robust methodologies to capture long-term behaviour and results associated with the programs. Future work should focus on evaluating a broader spectrum of programs such as doctoral- and post-doctoral-level experiential learning programs and underrepresented healthcare professions such as psychologists and dieticians. Integration of behaviour change and implementation science methodologies within the broader educational evaluation literature is also needed.

**Registry Name and Number:**

Open Science Framework, https://osf.io/5xqvg

## Introduction

 Post-secondary programs aim to prepare learners to use the theoretical knowledge and practical skills they have gained in the classroom to address evolving real-world challenges within a professional environment.^1^ Many post-secondary programs will enhance real-world learning opportunities through experiential learning and other educational approaches (eg, project-based learning, service learning).^1^ As an example, the Health System Impact Fellowship National Cohort Training Program (HSIF NCTP), funded through a grant from the Canadian Institutes of Health Research (CIHR) Institute for Health Services and Policy Research in 2019, is an experiential learning program that provides doctoral (PhD) students, post-doctoral fellows, and early career researchers the opportunity to become an embedded researcher within a health system organization.The HSIF NCTP was designed in response to escalating complexities within the healthcare sector by equipping learners with the requisite skills to navigate and address multifaceted real-world and professional workplace challenges.^2^ Aligned with this objective, the HSIF NCTP emphasizes cultivating a diverse set of enriched core competencies (eg, leadership, mentorship and collaboration, analysis and evaluation of health and health-related programs and policies; understanding and comparing healthcare systems and the policy-making process).^2^ These enriched core competencies generate standard professional development milestones for a cohort of trainees and scientists from diverse disciplines such as computer science, engineering, geography, medicine, nursing, occupational therapy, pharmacy, physiotherapy, and psychology. The educational themes of the HSIF NCTP are broadly centered around project management and leadership; patient-oriented research; equity, diversity, and inclusion; demonstrating impact through knowledge translation; building sustainability; and implementation science.^2^

 Many evaluations of educational programs focus on measuring knowledge acquired,^3-5^ but health education evaluations increasingly emphasize measurement of what is learned and practical application (ie, results) in the healthcare system (eg, Competency-Based Education^6^). The evolving emphasis on effective evaluation in health sciences education is driven by a myriad of pressing challenges and unique demands faced by healthcare professionals (eg, rapid technological advancements^7^; staffing shortages^8^; emphasis on interprofessional collaboration^9^; increasing healthcare service demand^10^). The dynamic landscape of healthcare systems, coupled with evolving patient needs and technological advancements, underscores the importance of knowledge- and skill-based continued education for healthcare professionals to ensure optimal patient outcomes and safety.^11^ Health sciences education is collaborative, where professionals from diverse disciplines contribute unique perspectives and expertise. This interdisciplinary environment presents both enriching opportunities and complexities within the teaching and learning environment. Amidst these dynamics, institutions are grappling with resource constraints and competing priorities.^12,13^ Educational activities are prioritized when they yield optimal outcomes aligned with learner and organizational goals and can enhance quality of patient care.^14^ Rigorous evaluation of educational programs is foundational for the judicious allocation of educational resources. Evaluating educational programs and interventions also ensures learners are equipped with the requisite attitudes, knowledge, and skills to contribute to the healthcare system workplace.^15-17^

 Evaluation frameworks (eg, Competency-Based Education^18^; Kern’s Six-Step Approach^19^; Kirkpatrick model^20^; Miller’s Pyramid^21^; Outcome-Based Education^22^) have emerged to assess the effectiveness of educational programs within health sciences education. These frameworks offer mechanisms for assessing educational programs, extending beyond mere knowledge acquisition to encompass practical application and impact across domains relevant to the healthcare sector. The Kirkpatrick model stands out as a widely adopted tool for evaluating educational training programs, particularly within post-secondary health sciences and related disciplines.^20,23-25^ The model delineates four levels (ie, *reaction*, *learning*, *behaviors*, and* results*) that each serve a distinct purpose in assessing program outcomes. At the *reaction* level, immediate learner responses provide insights into engagement and satisfaction, while the *learning* level evaluates the acquisition of knowledge, skills, and attitudes. The *behavior* level assesses the practical application of acquired knowledge in real-world scenarios, ensuring observable changes in behavior, and the *results* level, which presents the greatest measurement challenge,^26^ extends the model to encompass organizational outcomes, thus linking program success with broader organizational goals and objectives.^20,25^

 The popularity of the Kirkpatrick model stems from its comprehensive nature and adaptability, making it a valuable resource for evaluating program effectiveness and alignment with organizational goals. The model’s hierarchical structure posits a reinforcing relationship between its levels whereby outcomes at one level inform the next. For instance, favorable reactions are postulated to contribute to improved learning outcomes, thereby reinforcing the model’s relevance for institutional effectiveness and strategic planning.^20,25^ This integrated approach facilitates the identification of successful programs and potential unintended consequences of curriculum changes, ensuring strategic alignment. The model’s evolution, exemplified by the introduction of the New World Kirkpatrick Model in 2016, underscores its responsiveness to evolving demands, ensuring continued efficacy in evaluating and highlighting organizational impact across various programs.^20^ The Kirkpatrick model also has limitations. It assumes a linear progression across the four levels, potentially overlooking the dynamic and iterative nature of the education process, where feedback loops influence each level.^26,27^ The model’s emphasis on quantitative data may neglect the intricacies of educational outcomes, and its assumption of causal relationships between educational initiatives and outcomes may overlook contextual variables, such as prior knowledge or the organization, industry, or cultural environments.^26,28^ The model may inadequately address the evaluation of informal learning or on-the-job experiences, both of which significantly contribute to skill development.^28^ Variations in methodological approaches to its application have also led to limitations in the model’s effectiveness.^20^ Despite these limitations, the Kirkpatrick model remains the preferred option due to its practical applicability and versatility. The dynamic nature of healthcare systems focused on achieving optimal patient outcomes, necessitates rigorous evaluation of educational programs, highlighting the critical role of frameworks like the Kirkpatrick model.

###  Purpose and Research Questions

 We conducted a preliminary search of Cochrane Database of Systematic Reviews, Google Scholar, JBI Evidence Synthesis, Open Science Framework, and PROSPERO. Several systematic and scoping reviews of the use of the Kirkpatrick model across several disciplines (eg, business, nursing, physics) have been published,^29-31^ but none of these reviews examined the ways in which the Kirkpatrick model was specifically used to evaluate post-secondary health sciences programs. Thus, our purpose was to examine how the Kirkpatrick model is used in evaluating post-secondary health sciences programs to identify and analyze knowledge gaps. By identifying these gaps, we aimed to guide practices in evaluation studies. More specifically, our aim was to answer the following research questions: (1) What are the characteristics of studies using the Kirkpatrick model to evaluate post-secondary health sciences programs? (2) In which way has the Kirkpatrick model been applied to evaluate post-secondary health sciences programs? (3) What outcomes are associated with each of the Kirkpatrick model levels?

## Methods

 We conducted this scoping review using the Joanna Briggs Institute’s updated methodology for scoping reviews^32^ and the Preferred Reporting Items for Systematic Reviews and Meta-Analyses extension for scoping reviews (PRISMA-ScR).^33^ We chose a scoping review methodology because the primary purpose of this review was to consolidate existing literature, reveal patterns across existing literature, and identify gaps in the current literature, which was appropriate for our purpose given the anticipated diverse study designs and types of evidence that we would identify.^32^ The inherent adaptability of scoping reviews was seen as highly accommodating to the diverse study designs and types of evidence that were anticipated.^32^ We carried out our scoping review in several phases: (1) development of our review protocol and research questions, (2) systematic search for relevant studies, (3) study selection based on pre-established eligibility criteria, (4) data extraction, and (5) interpretation of results from the extracted data. Given the broader aims of scoping reviews,^32^ we did not perform a risk of bias or methodological quality assessment on the included studies. We registered our scoping review protocol with Open Science Framework (https://osf.io/5xqvg).

###  Search Strategy

 We developed a systematic search strategy with support from a librarian at the University of Regina (CB). We used the following search terms (ie, CINAHL, EMBASE, ERIC, MEDLINE, PsycINFO, Scopus): “Kirkpatrick evaluat*,” “Kirkpatrick model*,” “exp education, premedical” or “exp education, professional” or “exp schools, health occupations” or “exp universities,” “universit* or colleg* or postsecondar* or school* or educat* or residen*.” We completed the final search strategy was run on February 20, 2023 and February 21, 2023 (See Supplementary file 1).

###  Eligibility Criteria

*Time Period.* We included studies published in 2016 onwards and excluded studies published before 2016. We only considered studies published in 2016 onwards to only include studies using the most recent edition of the Kirkpatrick model.^20^ The most recent edition of the Kirkpatrick model comprises new constructs within each level, including engagement and relevance for the reaction level, confidence and commitment for the learning level, required drivers for the results level, and leading indicators for the impact level.

*Language.* We included studies published in English and excluded studies published in any language besides English. Evidence suggests that language constraints do not significantly impact review outcomes^34^; thus, our decision to exclude non-English studies presents minimal risk of bias.

*Publication Method.* We included peer-reviewed journal articles and books published through a scholarly press and excluded journal articles that were not peer reviewed, books published through a non-scholarly press, theses and dissertations, and conference proceedings.

*Study Design.* We included any empirical study using original data (eg, quantitative, qualitative, mixed methods, or case studies) and excluded non-empirical studies (eg, opinion pieces, commentaries) or studies not using original data (eg, reviews).

*Study Population.* We included studies with data on learners and/or educators and excluded studies with data on non-learners and non-educators.

*Education Setting.* We included studies examining programs within for-credit accredited university settings at the bachelor’s, master’s, doctoral, post-doctoral, post-graduate, or residency (eg, MD, PharmD) levels and excluded studies examining programs within non-credit, non-accredited, or non-university settings (eg, college diploma, post-graduate certification, Journeyperson certificate). The decision to focus on programs offering academic credit(s) aligns with our goal to ascertain the functions of the Kirkpatrick model when integrated into accredited university settings at all levels with structured courses and assessments.

*Educational Fields.* Our selection of educational fields was guided by the International Standard Classification of Education 2011.^35^ Educational fields that were eligible included education, humanities, social and behavioral science, journalism and information, business and administration, law, life sciences, physical sciences, mathematics and statistics, computing, engineering and engineering and trades, architecture and building, health, social services, environmental protection, and security services. Educational fields that were ineligible included manufacturing and processing, agriculture, forestry and fishery, veterinary, personal services, and transportation services.

*Evaluation Framework.* We included studies that reported on at least one level of the Kirkpatrick model (ie, *reaction, learning, behavior, results*)^20^ and excluded studies that did not report outcomes related to at least one level of the Kirkpatrick model. It was acceptable for studies to apply evaluation models in addition to the Kirkpatrick model.

###  Study Selection

**Figure 1:**
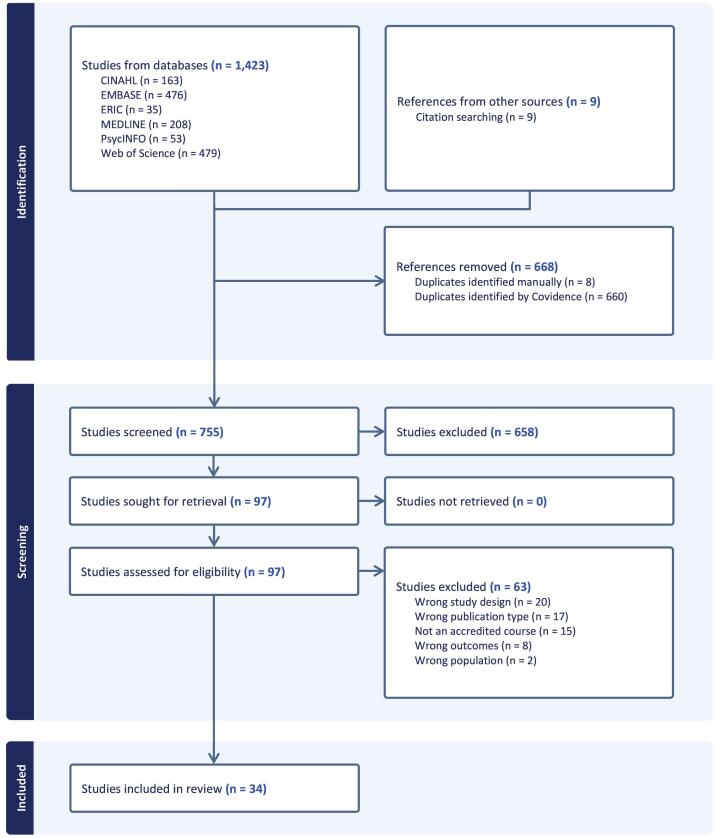
PRISMA-ScR Flow Diagram. Abbreviation: PRISMA-ScR, Preferred Reporting Items for Systematic Reviews and Meta-Analyses extension for scoping reviews.

 We uploaded all studies identified through our search strategy into Covidence.^36^ After Covidence automatically deduplicated the retrieved studies, two reviewers (BDR, CLR) independently screened the titles and abstracts and reviewed the full texts for the first ten studies to pilot the screening and reviewing processes. Following this pilot, the two reviewers discussed and reached a consensus regarding any changes to the screening and/or reviewing protocols. Next, the same two reviewers independently screened titles and abstracts and reviewed full texts for eligible studies. One of the reviewers (CLR) did a manual citation search of all eligible studies that resulted in the identification of nine additional studies that were considered at the screening stage. While reviewing full texts, the two reviewers documented reasons for exclusion. At the screening and reviewing stages, the two reviewers resolved disagreements through consensus. As shown in Figure 1, our search strategy yielded 1423 studies, of which 668 were duplicates, with 34 studies included in our scoping review.

###  Data Extraction

 Based on our scoping review’s research questions and with feedback from other authors, one of the authors (RJ) created a data extraction process in Google Forms. To pilot the data extraction process, two independent reviewers (CLR, BDR) extracted data from the first 10 studies. Following this pilot, the two reviewers discussed and reached a consensus regarding any changes to the data extraction process. One reviewer (CLR) extracted data for the remaining studies. Extracted data included authors, year of publication, study design, sample (learners or educators with, if appropriate, comparator groups), study purpose, education level, education field, education content, and the incorporation and definition of experiential learning and/or other educational approaches. We defined experiential learning as the process of learning through experience and through reflecting on this experience. We considered hands-on and related learning approaches as a form of experiential learning if it also encompassed learners reflecting on their work.^37^ Additionally, educational approaches that addressed the National Cohort Training Program core competencies were extracted. Evaluation models employed beyond the Kirkpatrick model, relevant study outcomes, and any programming adjustments proposed or implemented based on Kirkpatrick model outcomes were also extracted.

## Results

###  Study Characteristics

 We present a summary of study characteristics in Table.

**Table:** Study Characteristics

**Study**	**Year**	**Country**	**Study Design**	**Education Level**	**Education Field**	**Education Content**	**Sample**
^38^	2018	United States	Cross-sectional	PhD/Doctorate	Health sciences	Pharmacy	Learners, educators
^39^	2022	Turkey	Cross-sectional	Undergraduate	Health sciences	Medical education	Learners
^40^	2017	Sweden	Cross-sectional	PhD/Doctorate	Health sciences	Implementation science	Learners, comparator group
^41^	2023	United Kingdom	Cross-sectional	Continuing education	Clinical skills	Medical education	Learners
^42^	2021	United States	Cross-sectional	Undergraduate	Social sciences	Adaptive leadership	Learners
^43^	2020	Spain	Cross-sectional	Master’s	Health sciences, clinical skills	Palliative care	Learners, comparator group
^44^	2022	United States	Cross-sectional	PhD/Doctorate	Health sciences, clinical skills	Medical education	Learners
^45^	2021	United Arab Emirates	Cross-sectional	Undergraduate	Health sciences	Medical education	Learners
^46^	2021	Switzerland	Cross-sectional	Undergraduate	Clinical skills	Medical education	Learners
^47^	2019	United States	Cross-sectional	Master’s	Health sciences	Public health	Learners
^48^	2022	United States	Cross-sectional	PhD/Doctorate	Health sciences, clinical skills	Medical education	Learners
^49^	2020	United States	Cross-sectional	PhD/Doctorate	Clinical skills	Medical education	Learners
^50^	2019	Australia	Cross-sectional	Continuing education	Health sciences	Interprofessional clinical health service redesign	Learners
^51^	2020	India	Cross-sectional	Undergraduate	Health sciences, clinical skills	Medical education	Learners, educators
^52^	2021	United Arab Emirates	Cross-sectional	Undergraduate	Health sciences, clinical skills	Medical education	Learners, comparator group
^53^	2017	United States	Cohort	Master’s	Health sciences, clinical skills	Nursing	Learners
^54^	2022	United States	Cohort	PhD/Doctorate	Health sciences, clinical skills	Medical education	Learners, comparator group
^55^	2021	Canada	Cohort	Graduate or second-entry undergraduate	Health sciences	Interprofessional pain management	Learners
^56^	2016	Australia	Cohort	PhD/Doctorate	Health sciences	Interprofessional healthcare	Learners, comparator group
^57^	2022	India	Cohort	Undergraduate	Health sciences, clinical skills	Medical education	Learners, educators
^58^	2022	United States	Cohort	PhD/Doctorate	Health sciences	Medical education	Learners
^59^	2018	United States	Cohort	PhD/Doctorate	Health sciences	Medical education	Learners, educators, others
^60^	2021	Canada	Cohort	Undergraduate	Health sciences	Pharmacy	Learners, educators
^61^	2023	United States	Cohort	Master’s	Health sciences, clinical skills	Interprofessional geriatrics	Learners
^62^	2019	United States	Cohort	PhD/Doctorate	Health sciences	Interprofessional medication therapy management	Learners
^63^	2021	United States	Cohort	PhD/Doctorate	Health sciences, clinical skills	Medical education	Learners, educators, others
^64^	2021	United States	Cohort	PhD/Doctorate	Health sciences	Medical education	Learners
^65^	2018	Taiwan	Cohort	Master’s	Library or information science	Information organization	Learners, comparator group
^66^	2022	Italy	Mixed methods	Master’s	Clinical skills	Nursing	Learners
^67^	2022	Spain	Mixed methods	Undergraduate	Health sciences	Nursing	Learners, educators
^68^	2020	India	Mixed methods	Undergraduate	Health sciences	Medical education	Learners, comparator group
^69^	2022	Australia	Retrospective mono-centred	Undergraduate	Health sciences	Dentistry	Learners
^70^	2020	The Netherlands	Case series	Master’s	Health sciences	Interprofessional quality and safety in patient care	Learners
^71^	2021	United Arab Emirates	RCT	Undergraduate	Health sciences	Medical education	Learners, comparator group

Abbreviations: PhD, Doctor of Philosophy;RCT, randomized controlled trial. *Note.* Two studies did not clearly report on the education level of all study participants^56,62^

*Year of Publication.* The number of studies published increased each year with the peak number of studies published in 2021 (*n* = 9, 27%)^42,45,46,52,55,60,63,64,71^ and 2022 (*n* = 9, 27%).^39,44,48,54,57,58,66,67,69^ Only two (6%) studies were published in 2023,^41,61^ but we only ran the search strategy only a couple of months into 2023.

*Country.* Studies were predominantly conducted in the United States (*n* = 14, 41%).^38,42,44,47-49,53,54,58,59,61-64^ Studies conducted in other countries, including Australia,^50,56,69^ Canada,^55,60^ India,^51,57,68^ Italy,^66^ Spain,^43,67^ Sweden,^40^ Switzerland,^46^ Taiwan,^65^ The Netherlands,^70^ Turkey,^39^ United Arab Emirates,^45,52,71^ and United Kingdom,^41^ ranged from 1 to 3 studies.

*Study Design.* Most studies involved cross-sectional^38-52^ (*n*= 15, 44%)or cohort^53-63^ (*n*= 13, 38%)designs. Outside of these common designs, three studies (9%)employed a mixed methods design,^66-68^ one study (3%) was a retrospective mono-centered design,^69^ another study (3%) was a case series,^70^ and a final study (3%) was a controlled trial.^71^

*Education Level.* The sample included in each study was predominantly studying at the undergraduate level (*n*= 12, 35%)^39,42,45,46,51,52,57,60,67-69,71^ or PhD or other residency levels(eg, MD, PharmD; *n*= 12, 35%).^38,40,44,48,49,54,56,58,59,62-64^ Fewer studies evaluated master’s students (*n*= 7, 21%),^43,47,53,61,65,66,70^ continuing education (*n*= 2, 6%),^41,50^ or graduate and second-entry undergraduate trainees (*n*= 1, 3%).^55^ Two studies (6%) did not clearly report the education level of all participants in their sample.^56,62^

*Educational Field.* More than half of the studies incorporated health sciences curricula (*n*= 18, 53%),^38-40,45,47,50,55,56,58-60,62,64,67-71^ with four studies (12%) focused on clinical skills.^41,46,49,64^ Several studies addressed a combination of these topics(*n*= 10, 29%),^43,44,48,51-54,57,61,63^ while others covered topics related to social sciences (*n*= 1, 3%)^42^ or library and information sciences (*n*= 1, 3%).^65^

*Educational Content.* Half of the curricula were medical education (*n*= 17, 50%),^39,41,44-46,48,49,51,52,54,57-59,63,64,68,71^ while three studies (9%) exclusively assessed educational programming for nursing students.^53,66,67^ Several studies adopted an interprofessional approach, of which one study (3%) evaluated an implementation science curriculum^40^ and another study (*n*= 1, 3%) examined principles of adaptive leadership.^42^ Other interprofessional studies were oriented towards students across healthcare professions, and included themes such as clinical health service redesign (*n*= 1, 3%),^50^ pain management (*n*= 1, 3%),^55^ palliative care (*n*= 1, 3%),^43^ geriatrics (*n*= 1, 3%),^61^ public health (*n*= 1, 3%),^47^ and quality and safety in patient care (*n*= 1, 3%).^70^ One study (3%) explored interprofessional healthcare among allied health profession students^56^ and another (*n*= 1, 3%) focused on medication therapy management for multiple professions.^62^ Finally, one study (3%) evaluated a curriculum developed for dentistry students,^69^ while another study assessed an information organization course (*n*= 1, 3%).^65^

*Sample.* Most studies primarily focused on outcomes related to learners (*n*= 27, 79%)^39-50,52-56,58,61,62,64-66,68-71^ (of which eight studies [24%] included a comparator group)^40,43,52,54,56,65,68,71^ and five studies (15%) assessed both learners and educators.^38,51,57,60,67^ In addition, two studies (6%) involved individuals in supporting roles, such as clinical support staff, community partners, and program coordinators.^59,63^

###  Evaluation Methodologies for Kirkpatrick Model Levels

**Figure 2:**
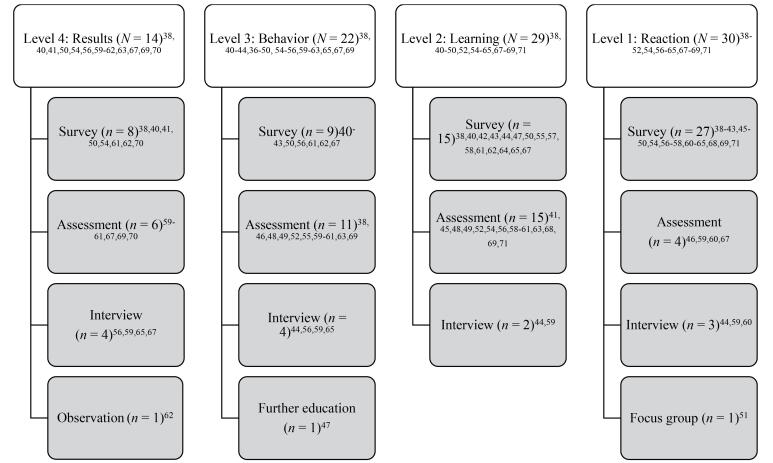
Number of Studies Using Specified Evaluation Methodologies Across Each of the Kirkpatrick Levels. *Note*. Some studies employed multiple evaluation methodologies per Kirkpatrick level.

 We present a summary of the inclusion of each of Kirkpatrick model levels along with evaluation methodologies used in each level in Figure 2. Of note, two studies (6%) in this review did not report how the chosen strategies were applied to each level of the model, resulting in unclear reported outcomes for each level. These studies similarly conducted interviews with participants (*n*= 1, 50%)^66^ or assessed outcomes through practical or written assessments or surveys (*n*= 2, 100%).^53,66^ Notably, another study combined analyses of behavior and results, making it unclear how to disentangle the findings from each level (*n*= 1, 3%).^41^

*Level One: Reaction. *Most studies assessed the initial reaction and engagement in the evaluated educational programs (*n*= 30, 88%),^38-52,54,56-65,67-69,71^ and primarily used self-report surveys (*n*= 27, 90%).^38-43,45-52,54,56-58,60-65,68,69,71^ Some studies conducted interviews with participants (*n*= 3, 10%),^44,59,60^ while others used formal practical or written assessments (*n*= 4, 13%).^46,59,60,67^ One study facilitated a post-course group discussion involving all faculty members engaged in the program (*n*= 1, 3%).^51^

*Level Two: Learning. *The second level of the model was assessed in almost all studies (*n*= 29, 85%).^38,40-50,52,54-65,67-69,71^ Of these, approximately half administered surveys to assess participant learning (*n*= 15, 52%),^38,40,42,43,46,47,50,55,57,58,61,62,64,65,67^ while others evaluated performance through written or practical assessments (*n*= 15, 52%).^41,45,48,49,50,54,56,58-61,63,68,69,71^ Two studies (7%) used participant interviews to address the second level of the model.^44,59^

*Level Three: Behavior. *Compared to levels one and two, fewer studies evaluated the behaviors level (*n*= 22, 65%).^38,40-44,46-50,54-56,59,60-63,65,67,69^ Similar to the previous two levels, behaviors were frequently evaluated via written or practical assessments (*n*= 11, 50%)^38,46,48,49,54,55,59-61,63,69^ or self-report surveys (*n* = 9, 41%).^40-43,50,56,61,62,67^ Select studies conducted interviews with participants (*n*= 4, 18%),^44,56,59,65^ while one demonstrated this level through students’ pursuit of additional learning outside the course (*n*= 1, 5%).^47^

*Level Four: Results. *The results level was the least frequently reported facet of the model, with just under half of the studies including it as an outcome (*n*= 14, 41%).^38,40,41,50,54,56,59-62,65,67,69,70^ Several of these studies measured impact through self-report surveys administered to participants in the training program (*n*= 7, 50%)^38,40,41,50,51,61,70^ or organizational participants (ie, the patients of healthcare professions students; *n*= 1, 7%)^62^ and slightly fewer used formal written or practical assessments (*n*= 6, 43%).^59-61,67,69,70^ Four studies (29%) incorporated interviews with participants for this level.^56,59,65,67^ One study conducted interviews with organizational informants and reviewed documents related to program outcomes (eg, newspaper articles on successful projects; *n*= 1, 7%),^56^ and one study employed informal observation of the organizational environment following program implementation (*n *=1, 7%).^62^

###  Outcomes Associated with Kirkpatrick Model Levels

*Level One: Reaction. *Across all studies that evaluated health sciences education above the undergraduate level (ie, continuing education, master’s, PhD or other doctorate programs), participant reactions demonstrate predominantly positive perceptions of the curricula, despite reporting several areas for improvement. For instance, students frequently regarded the courses as valuable, applicable, and satisfying (*n*= 14, 41%),^38,40,43,44,47,48,50,53,54,58,59,61,63,64^ reported that the content and formats were engaging (*n*= 2, 6%),^38,53^ and that the program would support their future work (*n*= 1, 3%).^50^ Some students appreciated sessions that encouraged collaboration and interaction with their colleagues (*n*= 2, 6%)^56,62^ and preferred sessions that facilitated active learning (*n*= 1, 3%).^62^ Others commented on the value of learning (*n*= 4, 12%) from their professors and mentors,^38,40,59,61^ as well as the enhancement of skill competence and confidence following the curriculum (*n* = 1, 3%).^48^ Studies that examined the perspectives of faculty or support staff yielded similar outcomes, as they also emphasized the importance of the mentor-mentee relationship (*n*= 1, 3%),^59^ perceived improvements in students’ skills and knowledge (*n* = 3, 9%),^38,59,63^ and found the courses engaging and useful (*n*= 1, 3%).^59^ Suggestions for improvement frequently pertained to the structure, format, and timing of the course. Participants expressed concerns about the course timing, tight timelines, and the demanding nature of the curriculum (*n*= 3, 9%),^38,50,54^ and prolonged gaps between sessions (*n* = 1, 3%).^58^ Additionally, other students noted a lack of support and clarity from instructors about course material and assignments (*n*= 3, 9%).^47,58,59^ Faculty and support staff expressed a need for improved processes in pre-work preparation (eg, grading, class presentations) and grading uniformity (*n* = 1, 3%)^38^ and mentorship support and communication (*n*= 1, 3%).^59^ Several studies noted participants’ desire for increased opportunities to apply their learnings in a real-world setting (*n*= 2, 6%).^40,53^

*Level Two: Learning. *All studies that assessed learning outcomes reported positive results for students, albeit to varying degrees. Most of these studies reported that the curriculum enhanced students’ learning and knowledge (*n*= 12, 35%).^38,40,43,44,47,48,50,53,54,56,62,65^ Several studies reported variable effects across their curricula. For instance, while Pfeifle et al found significant effects on learning within their program, they also noted high baseline scores, which may have limited the observable effects.^61^ In some cases, there was variability in skill acquisition, where students displayed increased confidence or knowledge, but this was not always reflected in their performance (*n*= 1, 3%),^58^ nor was it perceived as such by faculty (*n*= 1, 3%).^63^ Concerns about information retention and application were also highlighted. In these studies, some students expressed concerns about maintaining acquired knowledge over time (*n* = 1, 3%).^58^ This concern was echoed by community partners who suggested implementing practices to promote deeper learning (*n*= 1, 3%).^59^

*Level Three: Behavior. *At this level, reported outcomes indicated positive behavioral changes among participants. Results from course surveys demonstrated that many students either already had or expressed increased confidence in applying their acquired knowledge (*n*= 6, 18%).^40,43,47,50,53,59^ Analysis of examinations or assignments demonstrated behavioral changes pre- to post-curriculum (*n* = 1, 3%),^38^ as well as the achievement of course objectives (*n*= 1, 3%).^61^ Evaluation by instructors and observation of skill implementation suggested increased competency (*n*= 2, 6%)^48,59^ and perceived value of the achieved skills (*n*= 1, 3%).^44^ In one study, a comparison of standardized exam scores between a three-year and four-year cohort revealed no significant differences, suggesting the program’s effectiveness compared to the pre-intervention course (*n*= 1, 3%).^54^ Similarly, Sieplinga et al reported comparable results based on competency ratings by clinical support staff and attendings, showing no significant differences before and after the curricular intervention (*n* = 1, 3%).^63^ Additionally, surveys in one study indicated that patients were satisfied and had positive experiences with student teams (*n*= 1, 3%),^62^ while another study suggested that implementation of the new program successfully expanded and strengthened multidisciplinary learning experiences (*n*= 1, 3%).^56^

*Level Four: Results. *Studies frequently reported positive impacts of the training; however, identifying and implementing long-term impacts posed challenges. Some students perceived the course as complementing their learning (*n* = 1, 3%)^38^ and helping them obtain and improve skills (*n* = 3, 9%).^59,61,70^ Supporting staff and supervisors likewise identified the benefits of the program on students and their respective organizations (*n* = 2, 6%).^54,59^ One study assessed results through participants’ sharing of major projects (eg, conference presentation, publication, distributed outside their institution) and found that only 8% of participants did not share their work (*n* = 1, 3%).^50^ Another study distributed surveys to program directors to compare workplace-based performance outcomes between three-year and four-year medical degree students. The returned surveys demonstrated no significant differences between the cohorts, signifying that the two programs were comparable (*n* = 1, 3%).^54^ Craig et al found that there was some level of organizational improvements, such as continued use of a student project (eg, newly developed procedures in local healthcare services) following program cessation, in just over half of the interprofessional learning teams (*n*= 1, 3%).^56^ In addition, Schussel et al identified several organizational benefits of their program, including expanding allied professions’ roles, hiring a team of students following the program, and implementing additional staff training (*n*= 1, 3%).^62^ Despite indications of improvement, ascertaining the actual benefits of the programs are limited. Some students noted uncertainty about the long-term sustainability of programs (*n* = 1, 3%),^70^ or the authors did not collect patient outcomes or organizational data (*n* = 1, 3%).^61^ Alternatively, Carlfjord et al reported that while students believed the course content applied to their research, some mentioned it may not be as valuable for those in non-research-based settings (*n*= 1, 3%).^40^

###  Other Evaluation Models

 Seven (*n*= 7, 21%) of the studies integrated evaluation models in addition to the Kirkpatrick model, including the analyze, design, develop, implement, and evaluate (ADDIE) model;^45^ context, input, process, and product (CIPP) model^64^; Freeth/Kirkpatrick model^56,61,62^; and the strengths, weaknesses, opportunities, and threats (SWOT) model.^44,53^

###  Educational Approaches

**Figure 3:**
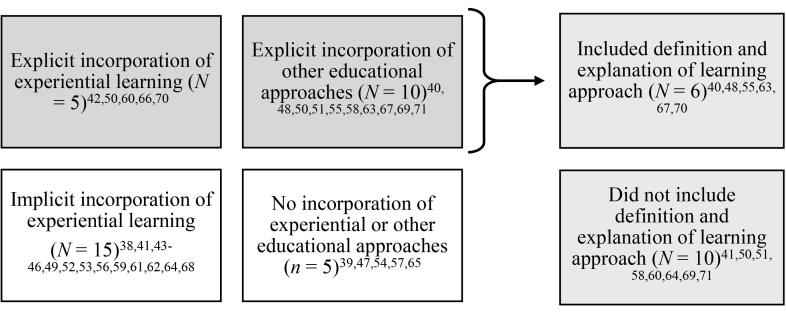
Number of studies incorporating and/or defining experiential or other educational approaches. *Note. *One Study Employed Experiential and Other Educational Approaches.^50^

 As shown in Figure 3, five studies (15%) explicitly incorporated experiential learning into their curriculum in some format,^42,50,60,66,70^ such as through the implementation of specialized projects (*n*= 1, 3%)^70^ or practical training sessions (*n *=1, 3%).^66^ Several studies explicitly incorporated other established educational approaches including constructivist (*n*= 2, 6%)^48,55^ or socio-constructivist learning theory (*n*= 1, 3%)^63^ and the Gibbs model of reflection (*n*= 1, 3%).^71^ Other explicitly incorporated educational approaches comprised of integrated problem (*n*= 1, 3%)^40^ or project-based (*n*= 1, 3%)^50^ learning, service learning (*n*= 1, 3%),^67^ and active learning (*n*= 3, 9%).^51,58,69^ Among the studies that explicitly incorporated experiential learning or other educational approach (*n*= 14, 41%),^40,42,48,50,51,55,58,60,63,66,67,69-71^ approximately half did not directly define the concept. Instead, many discussed its implementation broadly without directly describing the concept (*n*= 8, 57%).^42,50,51,58,60,66,69,71^ Nonetheless, six (43%) of the studies provided a clear definition and explanation of their respective framework.^40,48,55,63,67,70^

 A large proportion of the studies’ curricula described components of experiential learning without explicitly labelling them as being experiential (*n*= 15, 44%).^38,41,43-46,49,52,53,56,59,61,62,64,68^ The curriculum of five studies (15%) did not fit our criteria for experiential learning or other educational approaches.^39,47,54,57,65^

###  Enriched Core Competencies

**Figure 4:**
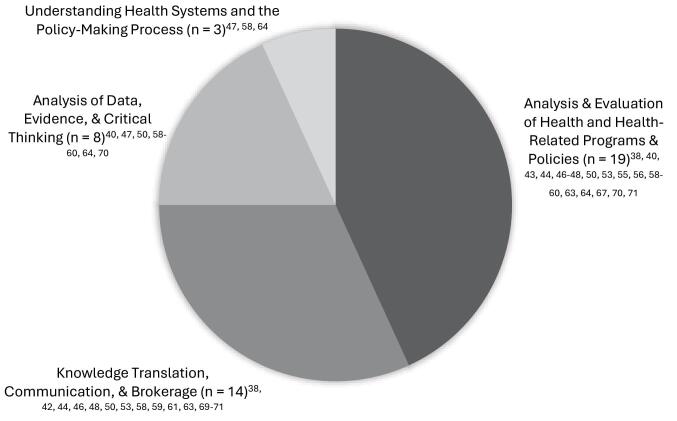
Number of Studies Incorporating Enriched Core Competencies Involving Research and Analytic Skills.

**Figure 5:**
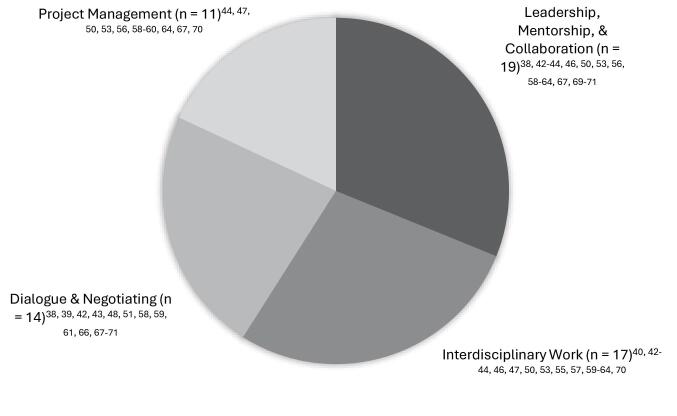
Number of Studies Incorporating Enriched Core Competencies Involving Professional Skills.

 As shown in Figures 4 and 5, studies frequently incorporated elements aligning with the recommended enriched core competencies outlined by the CIHR.^2^ A large proportion of these studies incorporated elements related to Research and Analytic Skills (*n* = 22, 65%).^38,40,42-44,46-48,50,53,55,56,58-61,62,64,67,69-71^ Even more studies incorporated elements related to Professional Skills (*n* = 27, 79%).^44,47,50,53,56,58-60,64,67,70^ Finally, 12 (35%) studies emphasized Change Management and Implementation,^40,42,47,50,53,55,56,58-60,64,70^ and six (18%) studies described Networking.^40,44,53,55,59,64^

###  Summary of Findings

 This scoping review examined the application of Kirkpatrick model in post-secondary health sciences education across each of the evaluation levels (ie, *reaction*,* learning*,* behaviors*, and *results*). Our first aim was to determine the characteristics of studies using the Kirkpatrick model to evaluate post-secondary health sciences programs. Most studies were conducted in the United States. The focus was primarily on undergraduate and professional students in fields like medicine and nursing, with less emphasis on graduate-level master’s and doctoral education. The educational content varied, with a focus on medical education, including specialized curriculum such as clinical skills and interprofessional programs.

 Our second aim was to understand the way in which the Kirkpatrick model has been applied to evaluate post-secondary health sciences programs. Our findings reveal a predominant reliance on self-report surveys. Most studies used cross-sectional or cohort designs, with some using mixed methods. Although not always explicitly defined, experiential learning was a significant focus of most studies. Some studies also incorporated additional evaluation models alongside the Kirkpatrick model (ie, ADDIE,^72^ CIPP,^73^ Freeth/Kirkpatrick,^74^ SWOT^75^).

 Our third aim was to identify outcomes of post-secondary health sciences programs associated with each of the Kirkpatrick model levels. The main findings of our scoping review suggest positive perceptions and outcomes associated with post-secondary health sciences programs under evaluation. Participants generally held favorable views of the curricula, citing their value, applicability, and engagement. Suggestions for improvement at the *reaction* level tended to focus on course structure and format. Learning outcomes were generally positive and perceived to be enhanced, although variations were observed across different subtopics within the curricula. Participants also reported positive *behavioral* changes, including increased confidence and skill competency. However, challenges were encountered in identifying and measuring the resulting *behavior *changes and long-term *results *of the programs.

## Discussion

 A notable pattern across the included studies was the reduced emphasis on Levels 3 (behavior) and 4 (results) relative to Levels 1 and 2.^76^ Although this has traditionally been attributed to practical barriers such as resource demands or methodological complexity,^76,77^ our findings suggest that conceptual and contextual mechanisms may also contribute to this pattern. Within the New World Kirkpatrick Model, meaningful movement from learning (Level 2) to behavior change (Level 3) depends on the presence of “required drivers,” including organizational reinforcement, supervisory support, and opportunities to apply newly acquired skills.^78^ Similarly, the ability to demonstrate results (Level 4) rests on the identification of “leading indicators” that serve as early signals of broader organizational impact. When these drivers and indicators are not intentionally built into training programs – or are not feasible to measure within academic timelines – evaluators may be limited in their ability to assess higher-level outcomes.^76^

 This mechanism-oriented interpretation aligns with broader evaluation and implementation science literature, which emphasizes that changes in professional behavior are shaped by the interaction between individual learning, contextual enablers, and organizational readiness.^79,80^ Consistent with this perspective, many programs in our review reported strong learning outcomes yet lacked corresponding structures in the workplace that would facilitate, reinforce, or monitor behavior change. Similarly, attempts to measure organizational results were constrained by challenges in accessing system-level data, attributing outcomes to a single educational intervention, and following learners long enough to observe sustained change. These contextual influences illustrate why higher-level outcomes are less frequently captured, even when programs appear to be well designed and positively received.^80^

 Taken together, these findings highlight the importance of designing evaluations that make mechanisms visible. Incorporating indicators such as learner confidence and commitment at Level 2, documenting environmental and supervisory supports at Level 3, and identifying feasible short-term organizational markers at Level 4 may enhance the quality and completeness of Kirkpatrick-aligned evaluations.^81^ In turn, these refinements can bridge the gap between what learners *know *and what they *do *in practice, supporting a more layered understanding of educational impact across levels.^79,80^

###  Strengths

 Our scoping review has several strengths. First, our use of the scoping review methodology allowed us to capture a wide range of studies relevant to evaluation of post-secondary health sciences programs, ensuring the inclusivity and breadth of our review, while also applying rigorous screening and selection procedures and meticulous data extraction approaches. Second, careful synthesis and analysis of the 34 eligible studies using the scoping review methodology allowed us to identify key themes, patterns, and gaps in broader literature. Third, the use of a scoping review methodology allowed for the inclusion of diverse study designs, methodologies, and educational contexts. Our review findings also highlighted several strengths of using the Kirkpatrick model to evaluate post-secondary health sciences programs. First, the Kirkpatrick model was used to evaluate a wide range of health sciences programs, demonstrating its adaptability across disciplines. Second, a considerable number of studies adopted a cross-sectional or cohort design. These methodologies are well-suited for assessing prevalence and relationships, either at a single point or over an extended timeframe, enabling an exploratory examination of training outcomes. A portion of the studies used a mixed-methods design, which, despite its increased complexity and demands in implementation, offers a comprehensive analysis by integrating quantitative and qualitative data. The review includes one study each of a retrospective mono-centered design, a case series, and a controlled trial. While the controlled trial is represented singularly, it brings a valuable experimental dimension to the predominantly observational nature of the studies included. Third, the explicit or implicit incorporation of experiential learning elements across the included studies implies a dynamic educational landscape, where educators are seen drawing from multiple strategies to enrich the learning environment. These findings suggest that educational programs in the health sciences are actively incorporating a broad spectrum of skills and competencies. This integration should equip learners to make meaningful contributions to learning healthcare systems. The studies placed significant emphasis on research and analytic skills, such as analyzing and evaluating healthcare programs and effectively communicating knowledge. Finally, the focus on interdisciplinary collaboration, effective communication, and adaptability highlights a forward-looking approach to professional development within the healthcare sector.

###  Limitations

 This scoping review was not without limitations. First, the scope and generalizability of the findings are limited, primarily stemming from the narrow focus on specific post-secondary health sciences programs. This narrow focus, particularly on nurses and doctors in terms of educational content, may inadvertently overlook the diverse educational needs of other health sciences programs, potentially constraining the generalizability of our conclusions across the broader spectrum of health sciences programs. Second, restricting the eligibility of our scoping review to post-secondary health sciences programs, rather than opening up our eligibility criteria to healthcare professionals already in the workforce, may limit the applicability of our findings exclusively to this specific cohort. Third, our focus on the Kirkpatrick model to the exclusion of other evaluation models (eg, ADDIE, CIPP) did not allow us to compare the suitability of these different models in evaluating post-secondary health sciences programs.

 Furthermore, with regards to our scoping review findings, several limitations were highlighted. First, our evaluation of educational outcomes reveals notable differences in assessing levels three (ie, *behavior*, 65% of eligible studies) and four (ie, *results*, 41% of eligible studies) compared to levels one (ie, *reaction*, 88% of eligible studies) and two (ie, *learning*, 85% of eligible studies), indicating a potential oversight in assessing the long-term impacts and practical applications of educational initiatives, which are critical for understanding the full spectrum of experiential learning’s benefits. Measuring *behaviors* or *results* may prove challenging due to several interrelated factors. Assessing the long-term effects of educational programs on individuals and organizations requires longitudinal studies, which are resource-intensive and time-consuming to conduct. Determining causality between educational programs and broader results, such as individual and organizational improvements, often involves accounting for multiple confounding variables. Thus, attributing observed results solely to educational programs amidst other concurrent initiatives or external factors adds complexity to the evaluation process. Second, our analysis of the identified studies revealed an overreliance on self-report surveys as the primary measure for evaluating each level. This indicates a heavy reliance on the perspectives of learners and educators to assess impact. While these perspectives are valuable, they inherently possess subjectivity and may introduce bias, potentially distorting the true impact of educational interventions.^82^ Third, our scoping review highlights the lack of studies examining program outcomes for post-doctoral fellows and very few studies examining outcomes for doctoral students in the health sciences, and this limitation is critical as findings derived from undergraduate programs may not generalize to doctoral and postdoctoral health sciences programs. Fourth, while the inclusion of interprofessional programs reflects the collaborative nature of healthcare, our findings suggest underrepresentation of certain healthcare professions within these curricula (eg, physical therapists, psychologists, social workers, dietitians). Fifth, a preponderance of studies originating from the United States means our review findings more readily generalize to this cultural context. Finally, theoretical limitations emerged from the absence of a standardized definition of experiential learning. The lack of a unified definition of experiential learning poses challenges in assessing its results and mechanisms within health sciences education.

 Because this review focused exclusively on studies using the Kirkpatrick model, our recommendations are intentionally situated within that framework. This approach reflects the model’s continued prominence in health sciences education and the need to strengthen how it is currently applied, rather than to compare it with alternative evaluation models.^83^ At the same time, the Kirkpatrick model has recognized limitations, including its assumed linearity and limited attention to contextual factors. For this reason, our recommendations emphasize ways to enhance Kirkpatrick-aligned evaluations by incorporating concepts from behavior change and implementation science, such as required drivers, leading indicators, and contextual enablers.^84^ These additions offer practical strategies to improve evaluation practice while preserving the scope and intent of this review. Future research would benefit from comparative work examining how the Kirkpatrick model performs relative to other evaluation frameworks in capturing mechanisms or change and longer-term outcomes.^83,85^

###  Future Directions

 Several future directions for our scoping review findings and scoping reviews more broadly merit consideration. First, future research needs to adopt longitudinal designs and include comparison groups to enhance the methodological rigor and validity of findings. These approaches could help determine causal relationships and observe educational outcomes for different groups over time for post-secondary professionals. Second, additional evaluation methods, such as interviews with participants or practical and written assessments, are needed to diversify the range of evaluation methods used. These supplementary measures enhance the depth and richness of the evaluation process, capturing not only participants’ perceptions but also their experiences and practical understanding of the educational content. Third, given the context-specific nature of our findings, it is essential for subsequent research to diversify the scope of programs evaluated, including various countries, cultural contexts, disciplines, educational levels, educational systems, and evaluation models. Fourth, we need clear definitions to facilitate a shared understanding of experiential learning among educators, researchers, and partners, promoting consistent and effective implementation of evaluation strategies. It also ensures more effective collaboration and knowledge exchange between partners. Fifth, given the collaborative nature of healthcare service delivery, future studies should explore an interprofessional approach to both education and evaluation, fostering interdisciplinary collaboration and enhancing the effectiveness of educational initiatives in post-secondary health sciences programs. Finally, theoretical advancements are also warranted, particularly regarding the integration of behavior change^86^ and implementation science^87^ literature into the conceptualization of the Kirkpatrick model. This integration would enable a more refined understanding of impacts across different evaluation levels.

 This scoping review was not without limitations. First, the scope and generalizability of the findings are limited, primarily stemming from the narrow focus on specific post-secondary health sciences programs. This narrow focus, particularly on nurses and doctors in terms of educational content, may inadvertently overlook the diverse educational needs of other health sciences programs, potentially constraining the generalizability of our conclusions across the broader spectrum of health sciences programs. Second, restricting the eligibility of our scoping review to post-secondary health sciences programs, rather than broadening our eligibility criteria to healthcare professionals already in the workforce, may limit the applicability of our findings exclusively to this specific cohort. Third, our focus on the Kirkpatrick model to the exclusion of other evaluation models (eg, ADDIE, CIPP) did not allow us to compare the suitability of these different models in evaluating post-secondary health sciences programs.

## Conclusion

 Collectively, our findings contribute to a more comprehensive understanding of applications of the Kirkpatrick model in accredited academic settings among interdisciplinary domains that support the healthcare system. Our findings revealed a range of applications of the Kirkpatrick model across various educational contexts with an emphasis on evaluating *reaction* and *learning* outcomes. While these findings provide insight into the immediate impacts of educational programs, a notable gap remains in evaluating *behaviors* and long-term *results*, which are crucial for program evaluation and subsequent improvements. Moreover, our results highlighted limited representation of certain healthcare professions and the absence of explicit definitions for experiential learning, suggesting areas for future research and practice development. Moving forward, educators and researchers are encouraged to adopt more robust evaluation methodologies in applying the Kirkpatrick model, including longitudinal designs, to better capture the full spectrum of outcomes associated with post-secondary health sciences education programs. Addressing these limitations can lead to improved quality and effectiveness in graduate-level health sciences education, ultimately advancing healthcare delivery and patient outcomes.

## Acknowledgments

 We would like to acknowledge Cara Bradley, Dr. John Archer Library & Archives, University of Regina, for her work on the initial and full search strategy for this manuscript. We also thank Briana De Roo for her assistance in screening titles and abstracts, reviewing full texts, and extracting data.

## Disclosure of artificial intelligence (AI) use

 Not applicable.

## Ethical issues

 Not applicable.

## Conflicts of interest

 Authors declare that they have no conflicts of interest.

## Supplementary files


Supplementary file 1. Search Strategies.
