# Supplementary material for: Applications of the Kirkpatrick Model in Post-secondary Health Sciences Education: A Scoping Review
Source: Int J Health Policy Manag. 2026 Apr 6;15:8857. doi: 10.34172/ijhpm.8857 (PMC13338747; doi:10.34172/ijhpm.8857)
Supplement: Supplementary file 1 — Search Strategies. [file ijhpm-15-8857-s001.pdf]

Article title: Applications of the Kirkpatrick Model in Post-secondary Health Sciences Education: A Scoping Review

Journal name: International Journal of Health Policy and Management (IJHPM)

Authors' information: Natasha L. Gallant[1], Elizabeth Oddone Paolucci[2], Chelsea L. Russill[1], Ray Jewett[3,4], Chantelle Recsky[5], Katherine Ford[6], Dina Idriss-Wheeler[7], Victrine Tseung[8], Hina Ansari[9], Zeenat Ladak[10], Aida Fernandes[11], Deborah A. Marshall[12]*

[1]Department of Psychology, Faculty of Arts, University of Regina, Regina, SK, Canada.

[2]Departments of Community Health Sciences and Surgery, Cumming School of Medicine, University of Calgary, Calgary, AB, Canada.

[3]Department of Geography and Planning, University of Toronto, Toronto, ON, Canada.

[4]Ontario Health, Toronto, ON, Canada.

[5]School of Nursing, Faculty of Applied Sciences, University of British Columbia, Vancouver, BC, Canada.

[6]Department of Kinesiology and Health Sciences, Faculty of Health, University of Waterloo, Waterloo, ON, Canada.

[7]Interdisciplinary School of Health Sciences, Faculty of Health Sciences, University of Ottawa, Ottawa, ON, Canada.

[8]School of Rehabilitation Science, Faculty of Health Sciences, McMaster University, Hamilton, ON, Canada.

[9]MAP Centre for Urban Health Solutions, Li Ka Shing Knowledge Institute, St Michael's Hospital, Toronto, ON, Canada.

[10]Department of Applied Psychology & Human Development, Ontario Institute for Studies in Education, University of Toronto, Toronto, ON, Canada.

[11]IMAGINE Network SPOR, McMaster University, Hamilton, ON, Canada.

[12]Departments of Community Health Sciences and Medicine, Cumming School of Medicine, University of Calgary, Calgary, AB, Canada.

***Correspondence to:** Deborah A. Marshall; Email: damarsha@ucalgary.ca

Citation: Gallant NL, Elizabeth Paolucci O, Russill CL, et al. Applications of the Kirkpatrick model in post-secondary health sciences education: a scoping review. Int J Health Policy Manag. 2026;15:8857. doi:10.34172/ijhpm.8857

Supplementary file 1. Search Strategies

Ovid MEDLINE(R) and In-Process, In-Data-Review & Other Non-Indexed Citations and Daily – February 20, 2023		
1	(Kirkpatrick adj3 evaluat*).mp.	135
2	(Kirkpatrick adj3 model*).mp.	314
3	1 or 2	360
4	exp education, premedical/ or exp education, professional/ or exp schools, health occupations/ or exp universities/	395,528
5	(universit* or colleg* or postsecondar* or post-secondar* or school* or educat* or residen*).mp.	2,092,131
6	4 or 5	2,095,369
7	3 and 6	254
8	limit 7 to yr="2017 - 2023"	208

CINAHL Plus (EBSCO) – February 20, 2023		
S9	S8 Limiters – English	163
S8	S3 AND S6 Limiters - Publication Date: 20170101-20231231	168
S7	S3 AND S6	259
S6	S4 OR S5	1,274,635
S5	universit* or colleg* or postsecondar* or post-secondar* or school* or educat* or residen*	1,270,357
S4	(MH "Education, Health Sciences+") OR (MH "Education, Clinical+") OR (MH "Education, Premedical") OR (MH "Colleges and Universities+") OR (MH "Schools, Health Occupations+")	350,880
S3	S1 OR S2	302
S2	Kirkpatrick N3 model*	234
S1	Kirkpatrick N3 evaluat*	202

EMBASE (Elsevier) – February 21, 2023		
#9	#8 AND [english]/lim	476
#8	#7 AND (2017:py OR 2018:py OR 2019:py OR 2020:py OR 2021:py OR 2022:py OR 2023:py)	479
#7	#3 AND #6	626
#6	#4 OR #5	25,151,654
#5	universit* OR colleg* OR postsecondar* OR 'post secondar*' OR school* OR educat* OR residen*	25,147,391
#4	'medical education'/exp OR 'paramedical education'/exp OR 'college'/exp OR 'medical school'/exp OR 'pharmacy school'/exp OR 'university'/exp	700,177
#3	#1 OR #2	649
#2	kirkpatrick NEAR/3 model*	509

#1	kirkpatrick NEAR/3 evaluat*	306

ERIC (Proquest) – February 21, 2023		
S1	Kirkpatrick NEAR/3 evaluat*	93
S2	Kirkpatrick NEAR/3 model*	95
S3	S1 OR S2	135
S4	MAINSUBJECT.EXACT("Health Occupations") OR MAINSUBJECT.EXACT("Medical Schools") OR MAINSUBJECT.EXACT("Allied Health Occupations Education") OR MAINSUBJECT.EXACT.EXPLODE("Medical Education") OR MAINSUBJECT.EXACT("Health Sciences") OR MAINSUBJECT.EXACT("Clinical Teaching (Health Professions)") OR MAINSUBJECT.EXACT("Premedical Students") OR MAINSUBJECT.EXACT("Medical Students") OR MAINSUBJECT.EXACT("Dental Schools")	26,311
S5	title(universit* or colleg* or postsecondar* or post-secondar* or school* or educat* or residen*) OR abstract(universit* or colleg* or postsecondar* or post-secondar* or school* or educat* or residen*) OR subject(universit* or colleg* or postsecondar* or post-secondar* or school* or educat* or residen*)	1,555,181
S6	S4 OR S5	1,555,433
S7	S3 AND S6	112
S8	(S3 AND S6) AND pd(20170101-20231231)	36
S9	(S3 AND S6) AND (la.exact("ENG") AND pd(20170101-20231231))	35

APA PsycINFO – February Week 2 2023		
1	(Kirkpatrick adj3 evaluat*).mp. [mp=title, abstract, heading word, table of contents, key concepts, original title, tests & measures, mesh word]	41
2	(Kirkpatrick adj3 model*).mp. [mp=title, abstract, heading word, table of contents, key concepts, original title, tests & measures, mesh word]	121
3	1 or 2	135
4	exp medical education/	26,388
5	exp Nursing Education/	7,238
6	exp Health Education/	20,749
7	exp higher education/	64,096
8	exp colleges/	19,129
9	(universit* or colleg* or postsecondar* or post-secondar* or school* or educat* or residen*).mp. [mp=title, abstract, heading word, table of contents, key concepts, original title, tests & measures, mesh word]	1,331,534
10	4 or 5 or 6 or 7 or 8 or 9	1,334,240
11	3 and 10	84

12	limit 11 to (english language and yr="2017 - 2023")	53

Web of Science (Clarivate) – February 21, 2023		
#9	#5 AND #6 and 2023 or 2022 or 2021 or 2020 or 2019 or 2018 or 2017 (Publication Years) and English (Languages)	479
#8	#5 AND #6 and 2023 or 2022 or 2021 or 2020 or 2019 or 2018 or 2017 (Publication Years)	491
#7	#5 AND #6	664
#6	universit* or colleg* or postsecondar* or post-secondar* or school* or educat* or residen* OR medical* OR dental* OR nursing OR allied OR clinical* OR premed* OR health* (All Fields)	41,019,409
#5	#3 NOT #4	726
#4	Sherrington-Kirkpatrick (All Fields)	658
#3	Search: #1 OR #2	1,291
#2	Kirkpatrick NEAR/3 model* (Topic) OR Kirkpatrick NEAR/3 model* (Title) OR Kirkpatrick NEAR/3 model* (Abstract)	1,157
#1	Kirkpatrick NEAR/3 evaluat* (Topic) OR Kirkpatrick NEAR/3 evaluat* (Title) OR Kirkpatrick NEAR/3 evaluat* (Abstract)	414
